# Screening for Early Gastric Cancer Using a Noninvasive Urine Metabolomics Approach

**DOI:** 10.3390/cancers12102904

**Published:** 2020-10-09

**Authors:** Hyuk Nam Kwon, Hyuk Lee, Ji Won Park, Young-Ho Kim, Sunghyouk Park, Jae J. Kim

**Affiliations:** 1 College of Pharmacy, Natural Product Research Institute, Seoul National University, Seoul 08826, Korea; hyuk.kwon@helsinki.fi; 2 Stem Cells and Metabolism Research Program, Faculty of Medicine/Helsinki Institute of Life Science, University of Helsinki, FIN-00014 Helsinki, Finland; 3 Department of Medicine, Samsung Medical Center, Sungkyunkwan University School of Medicine, Seoul 06351, Korea; leehyuk@skku.edu (H.L.); jeeewonpak@gmail.com (J.W.P.); younghokim@skku.edu (Y.-H.K.)

**Keywords:** gastric cancer, screening, metabolomics, urine

## Abstract

**Simple Summary:**

There are currently no effective specific biomarkers for the screening of early gastric cancer. Recently, metabolomics has been used to profile small endogenous metabolites, demonstrating significant potential in the diagnosis/screening of cancer, owing to its ability to conduct a noninvasive sample analysis. Here, we performed a urine metabolomics analysis in the context of an early diagnosis of gastric cancer. This approach showed very high diagnostic sensitivity and specificity and performed significantly better than the analysis of serum tumor markers modalities. An additional genomic data analysis revealed the up-regulation of several genes in gastric cancer. This metabolomics-based early diagnosis approach may have the potential for mass screening an average-risk population and may facilitate endoscopic examination through risk stratification.

**Abstract:**

The early detection of gastric cancer (GC) could decrease its incidence and mortality. However, there are currently no accurate noninvasive markers for GC screening. Therefore, we developed a noninvasive diagnostic approach, employing urine nuclear magnetic resonance (NMR) metabolomics, to discover putative metabolic markers associated with GC. Changes in urine metabolite levels during oncogenesis were evaluated using samples from 103 patients with GC and 100 age- and sex-matched healthy controls. Approximately 70% of the patients with GC (*n* = 69) had stage I GC, with the majority (*n* = 56) having intramucosal cancer. A multivariate statistical analysis of the urine NMR data well discriminated between the patient and control groups and revealed nine metabolites, including alanine, citrate, creatine, creatinine, glycerol, hippurate, phenylalanine, taurine, and 3-hydroxybutyrate, that contributed to the difference. A diagnostic performance test with a separate validation set exhibited a sensitivity and specificity of more than 90%, even with the intramucosal cancer samples only. In conclusion, the NMR-based urine metabolomics approach may have potential as a convenient screening method for the early detection of GC and may facilitate consequent endoscopic examination through risk stratification.

## 1. Introduction

Gastric cancer (GC) is the sixth most common type of cancer and the second leading cause of cancer-related mortality worldwide [1]. The overall survival and prognosis greatly depend on the disease stage, and the mortality from GC is mainly due to late presentation [2,3]. Therefore, early detection is critically important for reducing GC morbidity and mortality [4]. Further, early diagnosis is highly associated with a good prognosis [5,6,7]. Among screening methods for the early detection of GC, endoscopy is the most common modality [8]. However, endoscopy may be accompanied by complications, requires adequately qualified facilities, and is time-consuming [9,10,11,12,13,14].

The development of GC involves multiple genes and other factors, and tumors consist of mixed tissues and display various degrees of differentiation. There are currently no effective specific biomarkers for GC. Serum tumor markers are not satisfactory, mainly because of their poor diagnostic sensitivities. The sensitivities of Cancer embryonic antigen (CEA), CA 19-9, and CA 72-4 are <20% for an early stage and 20–50% for advanced stages of cancer [15,16,17,18]. Several studies have attempted to improve the diagnostic value of these markers by combining more than two markers or using adjusted cutoff values [19,20,21,22]. However, these markers remain unsatisfactory for GC diagnosis, especially at early stages. Currently, several biomarkers have been proposed for GC, including GLS/GGCT protein coexpression levels in tissue samples and circulating miR21 in serum samples [23,24]. Although these invasive methods have shown very satisfactory results with high sensitivities and specificities, it is still necessary to develop a reliable screening tool for the early detection of GC that is sensitive, specific, and easy to use; moreover, a mostly noninvasive approach is highly desired.

Metabolomics is used to profile small endogenous metabolites and has, in particular, demonstrated a significant potential in the diagnosis or screening of cancer, owing to its ability to noninvasively analyze samples [25,26,27,28,29]. Urine is arguably the most suitable sample because it is easy to obtain and can reflect the systemic metabolic status. Recently, urine metabolomics has been applied to the diagnosis of GC, with promising results [30,31,32,33,34]. A capillary electrophoresis-mass spectrometry (CE-MS)-based feasibility study by Chen et al. [30] used moving reaction boundary (MRB)-CE-MS for a better sensitivity and stability with a small number of samples. A nuclear magnetic resonance (NMR)-based metabolomics study [31] for early-stage GC diagnosis has also been reported; however, the study had multiple purposes, including the evaluation of curative surgery. Although the data showed great discrimination between groups based on urine sample analysis, the majority of the patient population had later than stage II GC. In other studies, the numbers of urine samples from patients with cancer and healthy controls were small (≤50), which could have limited the reliability of the studies. In addition, these studies included very limited numbers of cases of early GC, which is critical for GC screening [32,33,34]. Therefore, the actual diagnostic sensitivity and specificity for early GC detection by urine metabolomics have not been properly assessed.

In this study, we performed a urine metabolomics analysis for GC diagnosis, with a particular focus on early GC. The diagnostic performance of the metabolomics approach was evaluated with a rigorous cross-validation using a separate validation set. A relationship between metabolic contributors and the gene expression profile in cancer tissues was also studied.

## 2. Results

### 2.1. Subject Characteristics

The clinical information of the patients with GC and healthy subjects included in this study is detailed in Table 1. There were no significant differences in the demographic and laboratory findings between the two groups. The patients with GC were classified according to TNM (TUMOR-NODE-METASTASIS) staging as follows: stage I, 69 patients; stage II, 10 patients; stage III, 15 patients; stage IV, 9 patients. Thus, this study population included many more patients with stage I, particularly IA, than later-stage patients. There were 55 cases with differentiated and 48 cases with undifferentiated cancer. Lymph node metastases were found in 22 (21.4%) cases. The proportions of abnormal conventional serum tumor markers are also shown in Table 1.

### 2.2. Study Design

To evaluate the performance of the diagnostic model, validation was carried out with a separate validation set, as previously described [35]. Briefly, we randomly selected one-third of the samples from each group (within the cohorts) as the validation set (34 of 103 for the GC group and 33 of 100 for the healthy control group). The remaining samples (training set) were used to develop the diagnostic model. Similarly, in the analysis of the diagnostic performance of the three subgroups—stage IA cohort, stage I (IA+IB) cohort, and stage I+II cohort—one-third and two-thirds of the samples from each cohort were selected as the validation and training sets, respectively. These datasets from all cohorts were balanced for age, sex, Helicobacter infection, and other clinical characteristics (Table 1).

### 2.3. Discrimination between Urine Samples from Healthy Subjects and Patients with GC Using NMR Spectra

**Figure 1:**
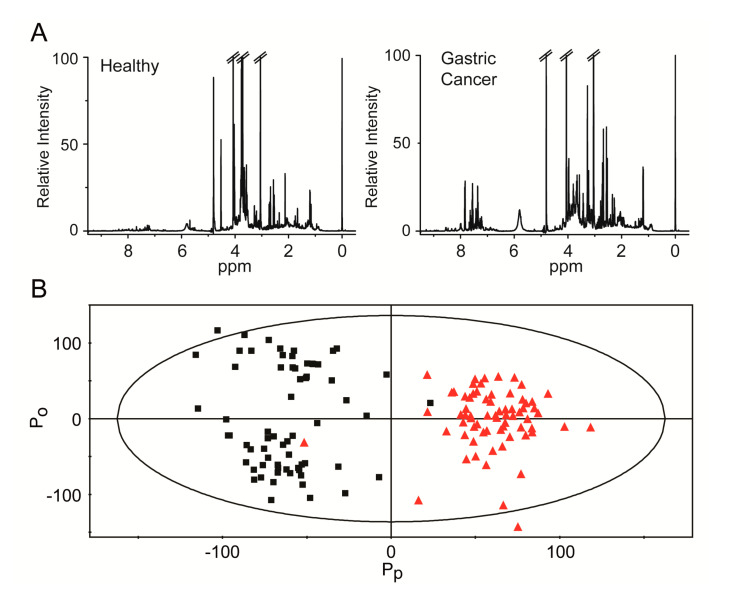
Representative ^1^H nuclear magnetic resonance (NMR) spectra of urine samples and discrimination between healthy control and gastric cancer groups. (**A**) Typical ^1^H NMR spectra with an internal standard signal (0.025% TSP) at 0 ppm. The left panel shows a representative ^1^H NMR spectrum for healthy controls, and the right panel shows a representative ^1^H NMR spectrum for patients with gastric cancer. (**B**) Orthogonal projections to the orthogonal projection for a latent structure-discriminant analysis (OPLS-DA) score plot of the healthy control and gastric cancer groups. The model was constructed with one predictive (*P_p_*) and two orthogonal (*P_o_*) components. The overall goodness of fit and cross-validation coefficients are 86.5 and 72.8%, respectively. Black boxes: samples from healthy controls; red triangles: samples from patients with gastric cancer.

All urine metabolic profiles, from both patients with GC and healthy controls, were obtained using NMR spectroscopy. Representative spectra from both groups were similar, but there were differences in specific regions, indicating subtle metabolic differences (Figure 1A). For a more holistic analysis of the NMR data for a training set, we performed a multivariate statistical analysis and established an orthogonal projection for a latent structure-discriminant analysis (OPLS-DA) model. The OPLS-DA approach is a method for the classification of groups with confounding factors, such as multicollinear and noisy variables [36]. The discrimination model was built with one predictive and two orthogonal components, and it exhibited an *R*^2^(*Y*) (overall goodness of fit) of 86.5% and *Q*^2^(*Y*) (overall cross-validation coefficient) of 72.8% (Figure 1B). Most of the samples in the prediction set were clustered into the corresponding groups, and only a few samples overlapped across the groups. In addition, a partial least squares-discriminant analysis (PLS-DA) score plot showed that there were no significant differences between the healthy subjects and GC patients at any stage based on *Helicobacter pylori* infection (Supplementary Materials Figure S1).

### 2.4. Analysis of Contributing Metabolites Using Statistical Total Correlation Spectroscopy (S-TOCSY)

The identities of the metabolites that contributed to the difference between the cancer and healthy control groups were determined by assigning NMR peaks and performing a statistical analysis (Table 2, Figure S2).

**Figure 2:**
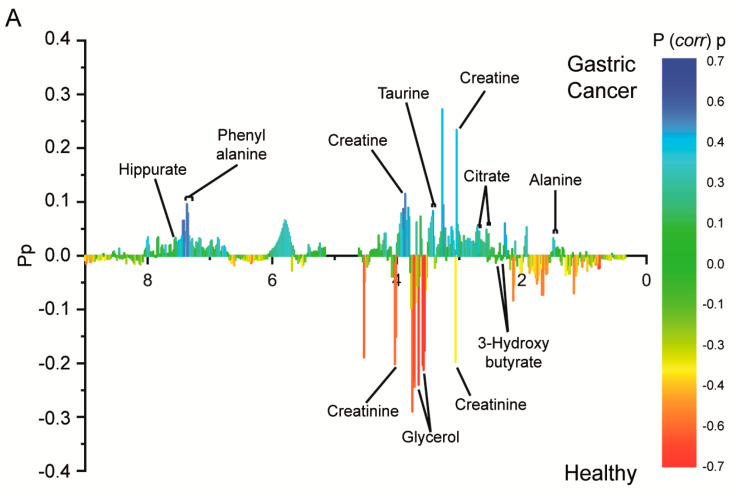

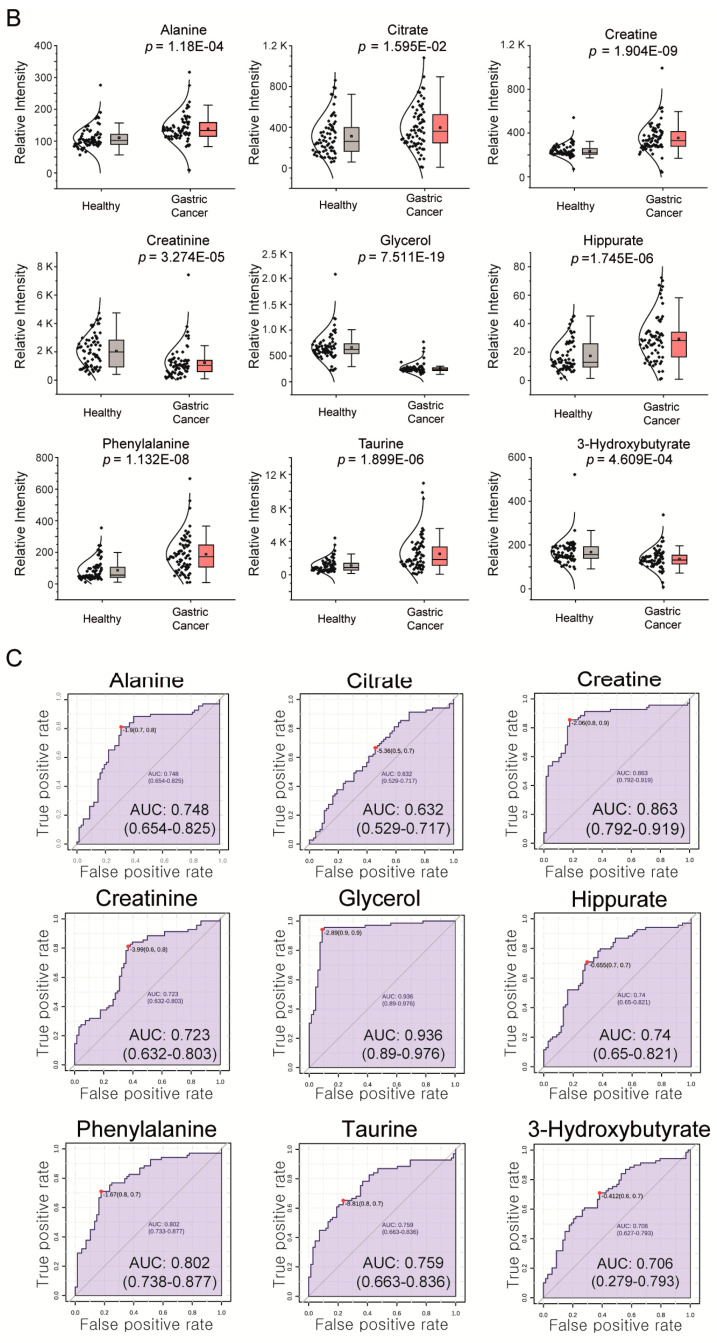
Identification of contributing metabolites by statistical total correlation spectroscopy, their relative levels in urine samples, and ROC analysis. (**A**) The color scale P(corr)p on the right represents a modeled correlation, and Pp represents a modeled covariation. The names are indicated for metabolites with noticeable signals, whose distribution was different between the healthy control and gastric cancer groups. (**B**) Relative concentrations of identified metabolites in urine samples from healthy controls and patients with gastric cancer and associated *p*-values. The shown false discovery rate (FDR)-adjusted *p*-values were acquired through the online statistical analysis tool Metaboanalyst (www.metaboanalyst.ca). (**C**) ROC curves of each identified metabolite, with area under the curve (AUC), sensitivity, and specificity.

All metabolites with statistically meaningful differences were identified by matching their peak characteristics with those in databases and are shown in Figure 2A and Table S1. Of the identified metabolites, alanine, taurine, phenylalanine, and creatine have been previously described as urinary metabolomics markers for GC [31,32,33,37]. We observed low creatine and high creatinine levels in the urine from the normal subjects and, conversely, high creatine and low creatinine levels in the urine from the patients with GC (Figure 2B). The levels of these markers varied rather widely, with those of citrate showing the widest variation between the healthy control and cancer groups (Figure 2B). In addition, the fold increases were not very large, and thus it was difficult to find a single representative marker metabolite. As the (P *corr*) *p*-values of each signal in the S-TOCSY plot were also rather small (maximum value ≤0.7), multiple metabolites, rather than one or two major metabolites, seemed to intricately contribute to the group separation. Additionally, we performed a receiver operating characteristic (ROC) curve analysis, and the results showed a broad range of areas under the curves (AUCs), from 0.632 (citrate) to 0.936 (glycerol), with varying sensitivities and specificities (Figure 2C). The results of the ROC analysis are summarized in Table 2.

### 2.5. Diagnostic Performance: Validation of the Prediction Model

We developed a prediction model that discriminated between cancer and healthy control samples using a training set. Therefore, we tested whether our model could predict the cancer status of unknown samples from the validation set, which were set aside during model building.

**Figure 3:**
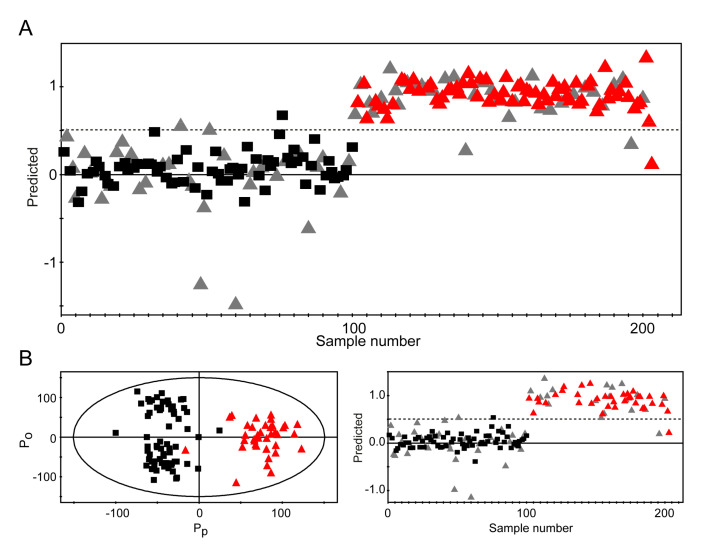
Prediction of healthy subjects and patients with gastric cancer using unknown samples and early-stage diagnosis. (**A**) For validation, 67 unknown samples (33 healthy control and 34 gastric cancer samples) were fitted into the OPLS-DA model. The class membership of the unknown samples was predicted using an a priori set cutoff value of 0.5 (dashed line). The *Y*-values of the black boxes and red triangles are from the analysis of known samples (67 healthy control and 68 gastric cancer samples), and gray triangles represent unknown samples. (**B**) For early-stage diagnosis, the gastric cancer group only comprised patients with stage IA cancer (*n* = 37), and the diagnostic model was established using the same healthy control group (*n* = 67). Validation was performed with 52 unknown samples (gray triangles). Black boxes: samples from healthy controls; red triangles: samples from patients with gastric cancer. In the case of misclassified samples, the predicted *Y*-values for unknown samples are shown as gray triangles.

NMR data of the validation set were obtained using the same experimental parameters as those used for the training set, and the data were fitted into the prediction model to obtain the cancer status using an a priori set cutoff value of 0.5 for the predicted dependent variable (Figure 3A).

This validation test correctly predicted the respective status of 31 of 33 healthy control samples and 32 of 34 cancer samples, thus yielding a specificity of 93.9% and a sensitivity of 94.1% for the diagnosis of GC. Moreover, the sensitivity and specificity of CEA, CA19-9, and CA72-4 were determined (Table 3). Compared with the serum tumor markers for the same patients, the metabolomics approach performed significantly better, especially in terms of sensitivity.

For early GC screening, it is desirable to have a diagnostic model that can perform well without the inclusion of later-stage samples. Therefore, we carried out a diagnostic performance test as above after excluding later-stage samples (Figure S3 and Table 3). The metabolomics approach showed a robust performance even with the earliest, stage IA samples (*R*^2^ > 0.508 and *Q*^2^ > 0.676) and exhibited a specificity of 97% (32 correct predictions for 33 healthy control samples) and a sensitivity of 94.7% (18 correct predictions for 19 cancer samples) (Figure 3B).

### 2.6. Relationship between Urine Metabolites and Gene Expression in Cancer Tissues

After confirming the utility of the proposed model in predicting early GC, we wanted to determine whether there was a relationship between the metabolic contributors and gene expression profiles in GC. Although one or two significant marker metabolites did not account for a large part of the metabolic alterations between the healthy control and cancer groups, a metabolic–genetic relationship might reveal a weak but significant correlation. To this end, we analyzed microarray data for patients with GC (*n* = 103) and normal controls (*n* = 29) and examined the expression levels of genes known to metabolize the identified contributing metabolites [38]. We applied the bioinformatics network visualization tool MetScape to perform a pathway-based network analysis [39]. The software allows the visualization and interpretation of metabolomics and gene expression datasets in a human metabolic network context. Using the compound–reaction–enzyme–gene analysis function of MetScape, we could obtain enzymes potentially related to the metabolites identified by our metabolomics analysis. In brief, we conducted a pathway analysis based on our metabolomics results to elucidate potentially related genes (Figure S4A). Subsequently, we analyzed the expression levels of the genes encoding these enzymes using the Chen’s microarray dataset and found significant changes in the expression of some genes (Figures S5 and S6; Table S3). We performed a volcano plot analysis for selecting meaningful genes based on a fold change of 1.5 and a false discovery rate (FDR) of 0.005. As a result, we selected five genes including *ACLY*, *ACO2*, *BAAT*, *CKMT1B*, and *GGTL4* (Figure S4B). A joint pathway analysis using the identified metabolites and correlated genes was also performed; overall, the result supports the abovementioned findings (Figure S4C).

## 3. Discussion

Metabolomics, which is used to assess the overall metabolic profiles of biological samples, may help establish the missing link between gene/protein expression profiles and final cellular phenotypes in normal and diseased states. Metabolomics urine analysis is also attractive for the routine monitoring of cancer because urine contains high concentrations of many water-soluble metabolites present in plasma and sampling is noninvasive. Several studies have shown that urine metabolomics analysis provides a potential diagnostic tool for the early detection of cancer [27,40,41,42].

To date, several studies have explored the utility of metabolomics in the diagnosis of GC. Early reports mostly used tissue samples obtained from surgery or endoscopic biopsies [37,43,44], aiming at directly detecting metabolic alterations in cancer tissues. However, studies that are more recent have obtained comparable results using more accessible samples such as blood or urine [31,34,45]. Important drawbacks of these studies were small numbers of cases and a lack of rigorous cross-validation to assess the actual diagnostic performance of the metabolomics approach. The current study has thus far employed the largest number of cases. In addition, our validation set corresponded to one-third of all enrolled cases and was larger than those used in most of the previous studies. Therefore, the high diagnostic performance of our approach should be considered more reliable than that of previous studies.

Clinically, another equally important aspect of our study was the inclusion of a large number of patients with stage I GC. Among the enrolled cases with cancer, more than half had stage I GC, including 56 cases with intramucosal cancer, which can be treated by endoscopic resection. In addition, we achieved a comparably high diagnostic performance using only intramucosal lesions. In comparison, a previous study that reported the diagnostic performance of metabolomics included no stage IA cases in the training set, although it included 13 stage IA cases in the validation set [31]. It should be also noted that conventional tumor markers performed very poorly, with a low sensitivity. The current study is the first noninvasive metabolomics diagnosis study that focused on early GC.

Despite the high diagnostic performance, our approach could not establish a firm mechanistic link between metabolite changes and GC, nor could it identify a single marker metabolite that explained the metabolic difference between healthy controls and cases with GC. Nevertheless, this analysis can be performed quickly and affordably, provided a diagnostic facility is available, at a low cost and without the need for medically trained personnel. Therefore, the urine metabolomics approach should be suitable for early GC screening, rather than the confirmation of GC, which should be performed by expert pathologists. Combined with the convenience and noninvasive nature of urine analysis, this method should be applicable to the general population.

Although we found several metabolites that contributed to the metabolic difference between patients with cancer and healthy controls, the correlations of these metabolites with differences between the groups were rather small. Interestingly, we found that the creatine and creatinine levels showed opposite trends in the normal controls and in patients with GC. Up to 94% of creatine is found in muscle tissues, and it is nonenzymatically converted to creatinine and excreted into urine through the kidney [46]. A significant correlation has been reported between creatine metabolism and muscle mass [47], which is helpful in interpreting the inverse relationship between the measured concentrations of these two metabolites in our study. Compared with that in normal controls, muscle mass is likely to be relatively deteriorated in patients with GC, which may lead to abnormal creatine metabolism. Based on these effects, the high levels of creatinine were interpreted to be due to the conversion of creatine to creatinine in the normal controls, while relatively low levels of creatinine were measured in the patients with GC. Creatine conversion to creatinine is a nonenzymatic reaction, which is potentially due to the failure to find specific enzymes associated with this reaction.

Unfortunately, with insufficient metabolic data, a direct mechanistic investigation of GC was not possible in the current study. However, the diagnostic performance was evaluated using all the metabolite signals as a whole and was found to be much higher than that of conventional markers or a radiological approach. In fact, one advantage of the metabolomics approach is that no identification of a single significant marker is necessary for disease diagnosis, as noted previously [48]. In addition, it has been noted that the measured metabolite profile is a reflection of the convergence of tumor, microenvironment, and global metabolic alterations [49]. Therefore, finding one or two significant metabolites whose levels can account for a large part of metabolic differences between patients with cancer and normal cases seems to be very difficult, if possible. Actually, there are noticeable variations in the levels of contributing metabolites. These variations may reflect the well-known heterogeneity of solid tumors. In a large-scale sequencing analysis of major human cancers, it was estimated that 3000 to 10,000 mutations could be found in one of the cancer genomes [50]. This heterogeneity may account for the difficulties in finding a single significant metabolite marker.

Citrate, a metabolic intermediate of the tricarboxylic acid (TCA) cycle, has been previously identified as a GC biomarker [51], but there remain inconsistencies regarding its levels, with some studies reporting increases and others reporting decreases in citrate levels in GC [51,52]. These differences may indicate that the distribution range of citrate levels is quite large in GC, as was observed in our study. However, further qualified studies, including a controlled trial or a cohort study, should be conducted to validate the screening programs using urine metabolomics profiles.

Although our metabolomics results showed different total metabolic profiles between healthy and GC patients, these results cannot stand alone. The complementation for functional linkage via the incorporation of other “omics” datasets in the context of system biology is always required. For this purpose, we conducted a pathway analysis using microarray data to disclose any potential correlation with our metabolomics results. Importantly, we found several metabolite-gene expression correlations. For instance, the expression of *ACO2*, an enzyme that plays a critical role in the catalysis of citrate to isocitratewas down-regulated in GC patients, supporting the increased citrate levels observed through our metabolomics analysis. Moreover, *GGTL4*, a member of gamma-glutamyltransferase that plays a key role in the transfer of gamma-glutamyl functional groups to amino acids, expression levels were down-regulated in GC patients. Although a direct correlation between the *GGTL4* levels and the production of alanine and taurine may be questionable, *GGTL4* is located in a metabolic pathway close to those essential for the production of these metabolites. Another gene responsible for the catalysis of acyl-CoA thioester into either glycine or taurine, *BAAT*, was up-regulated in GC patients; again, this could be the reason for the elevated taurine levels in GC patients. We also found that mitochondrial creatine kinase (*CKMT1B*) was differentially expressed in GC patients. CKMT1B is responsible for the transfer of high-energy phosphate groups from the mitochondria to creatine. However, this gene is not directly involved in creatine production, making it difficult to determine the correlation between the expression of *CKMT1B* and the creatine levels in GC patients. Importantly, overall, we confirmed that the suggested metabolite-gene correlation was supported by the results of additional joint pathway analysis.

In addition, the pepsinogen I/II ratio, which is recently coming into the spotlight, for identifying a high-risk GC group was not examined in this study. However, this biomarker is known to be a useful serologic marker for chronic atrophic gastritis, which is a precancerous lesion. Hence, the purpose of this marker is not consistent with this study as this study demonstrated the diagnostic performance of a metabolomics profile in the detection of GC regardless of accompanying atrophic or metaplastic change. Furthermore, as the sensitivity and specificity of the pepsinogen test differ from country to country, further research is required to increase the efficacy of pepsinogen as a GC biomarker [53]. Another issue is about change of metabolomics profile after treatment. In our study, we only compared active cancer patients and healthy subjects. The evaluation of the urine metabolomics profile of gastric cancer patients, after curative resection, should also be considered, even though there could be a confounding bias due to the dietary life change after surgical gastrectomy. This said, further studies are warranted to elucidate the metabolomics change in gastric cancer subjects, before and after surgery.

For the NMR spectra normalization, we applied the “total area normalization approach”, a numerical normalization method rather than a physiological method using, e.g., the osmolality or creatinine levels. Indeed, the normalization against creatinine should be used when the creatinine clearance is constant under stable metabolic regulation. Of note, such an approach is not suitable for our study, because gastric cancer is one of the many diseases causing metabolic dysregulation. Even though the urinary specific gravity and osmolality measurements are currently recommended as normalization methods for the analysis of urine samples, these approaches require an additional measurement, a critical limitation in the context of high-throughput screening research. While various numerical normalization methods are proposed, each approach has its own advantages and limitations [54]. Therefore, the standardized normalization method for NMR-based urine metabolomics has not been established. However, for large sample size-based studies (over 50 samples), the quantile normalization has been suggested with better results, which we will consider as an additional option in further research.

## 4. Materials and Methods

### 4.1. Patients

The study design was approved by the Institutional Review Board at the Samsung Medical Center (IRB No. 2012-08-045), and written informed consent was obtained from all subjects enrolled in this study. We collected urine samples from patients with GC (*n* = 103) and age- and sex-matched healthy subjects (*n* = 100) at the Samsung Medical Center. Patients with prior treatment, including chemotherapy or surgery, serious complications such as active bleeding or an obstruction, abnormal liver function or renal function test results, severe cardiopulmonary disease, collagen disease, uncontrolled diabetes mellitus, and active carcinoma at other sites were excluded. All patients were diagnosed using biopsy or surgical resection. Healthy controls were age- and sex-matched subjects with no declared history of any gastrointestinal or chronic diseases, and no gastrointestinal symptoms. Furthermore, healthy controls showed normal endoscopic findings, as per the endoscopic examination results. Subjects diagnosed with benign gastric diseases including gastritis, gastroesophageal reflux disease, ulcer, or benign tumors (as per the endoscopic examination results) were not included in this study. Control samples were obtained from healthy subjects who underwent a routine health check-up at the Samsung Medical Center in the same period. The selection criteria required individuals to not meet the above-listed exclusion criteria, including neoplasms. All subjects were instructed to fast for 8 h before the collection of urine samples. Participants provided a midstream urine sample. No dietary restriction or activity modification was required before the urine collection.

### 4.2. Serum Assays of CEA, CA 19-9, and CA 72-4

Blood samples were obtained from all patients in the morning during the week before surgery. The blood sample was centrifuged at 1000× *g* for 10 min to separate the plasma from blood cells. Serum CEA, CA 19-9, and CA 72-4 were measured using a radioimmunoassay. The normal values of CEA, CA 19-9, and CA 72-4 were set at less than 7 ng/mL, 35 U/mL, and 4 U/mL, respectively.

### 4.3. Urine Sample Collection and Preparation

First morning urine samples (3–5 mL) were collected and centrifuged at 3000 rpm for 15 min at 4 °C. The supernatants were transferred to frozen tubes and stored at −80 °C until processing. The frozen urine samples were gently thawed and centrifuged at 15,000 rpm for 20 min at 4 °C. A 500-µL aliquot of the supernatant was mixed with 50 µL of phosphate buffer (1.5 M K_2_HPO_4_, 1.5 M Na_2_HPO_4_, and pH 7.4), centrifuged at 15,000 rpm for 20 min at 4 °C, and then incubated at room temperature for 10 min. For the internal standard, 50 µL of a 0.25% trimethylsilylpropanoic acid (TSP) solution in D_2_O was added to 450 µL of a centrifuged supernatant, and the mixture was transferred into a 5-mm NMR tube.

### 4.4. NMR Data Acquisition and Determination of Metabolic Profiles

All ^1^H NMR spectra were obtained using a 500-MHz NMR spectrometer (BioSpin Avance 500; Bruker, Billerica, MA, USA), which was operated at a 500.13-MHz proton frequency at 25 °C using the Carr–Purell–Meiboom–Gill pulse sequence (cpmgpr1d) with 400 ms of the total spin echo delay. The acquisition parameters and data processing steps were as reported previously [35,55]. In brief, a proton spectral FID was collected within a 14-ppm spectral width during 128 scans. A Fourier transformation and phase correction were applied to proton NMR signals using the MNova software (Mestrelab Research S.L., Escondido, CA, USA). The water signal region (4.62–5.15 ppm) was excluded, and the data were normalized and referenced to the total area integration values and the 0.025% TSP value, respectively. All the processed data were compared against the Chenomx NMR database version 8.2 (Spectral Database, Edmonton, AB, Canada) to identify representative metabolites by shifting in appropriate pH ranges. To distinguish between the healthy controls and patients with GC, OPLS-DA was performed using SIMCA-P version 11.0 (Umetrics, Umeå, Sweden) by applying spectral binning data by using an in-house Perl script with a 0.0073-ppm width. To evaluate the performance of the OPLS-DA model, validation was carried out using a separate validation set. Briefly, one-third of the samples from each cohort were randomly selected and used as the validation set using the k-fold cross-validation method. The diagnostic performance of the OPLS-DA model was evaluated based on the Y-variable values of the validation set, obtained using the training set model with an a priori set cutoff value of 0.5. The sensitivity and specificity values were obtained based on the correct prediction of the 33 healthy control and 34 GC patient samples during the validation step. All statistical analyses were performed using statistical software and an online data analysis tool, including SIMCA-P version 11.0 (Umetrics, Umeå, Sweden), OriginPro 8 (OriginLab Corporation, Northampton, MA, USA), R (The R Foundation for Statistical Computing, Vienna, Austria), and Metaboanalyst (www.metaboanalyst.ca).

### 4.5. Microarray Data Analysis

The variation of gene expression in human GC was investigated using the microarray results by Chen et al. [38] Genes involved in the metabolism of the contributing metabolites, identified by S-TOCSY, and were investigated for their differential expression in cancer and normal samples. In detail, we performed pathway-based network analysis using MetScape to visualize and interpret metabolomics and gene expression data in the human metabolic network [39]. We used metabolites identified by our metabolomics analysis, and the pathway-based network was built using the compound–reaction–enzyme–gene method. Subsequently, we obtained the names of the genes potentially associated with the input metabolites and analyzed the gene expression levels from Chen’s microarray dataset [38]. From Chen’s research dataset, we downloaded all individual microarray data from normal control and patients, and generated a new summarized gene list consisting of “R/G Normalized (Mean)” value to determine the fold changes and adjusted *p*-values of each gene.

## 5. Conclusions

In conclusion, we report here a metabolomics approach for GC screening using urine samples from patients who were mostly diagnosed with early GC and healthy subjects. This approach showed very high diagnostic sensitivity and specificity using a validation set and performed significantly better than did serum tumor markers modalities. An additional genomic data analysis revealed an up-regulation of the expression of several genes in GC. As this was the largest metabolomics study and the first that mostly focused on early GC, the approach developed may have the potential for a mass screening of an average-risk population and may facilitate endoscopic examination through risk stratification.

## Figures and Tables

**Table 1:** Clinicopathologic characteristics of the study participants.

Variables	Whole Training Set			Whole Validation Set		
	Gastric Cancer (*n* = 69)	Control (*n* = 67)	*p*-Value	Gastric Cancer (*n* = 34)	Control (*n* = 33)	*p*-Value
Age (years), mean (SD)	53.3 ± 11.1	54.9 ± 11.8	0.447	54.1 ± 9.1	55.4 ± 10.2	0.519
Gender, male (%)	68.1	71.6	0.654	58.8	66.7	0.507
BMI, mean (SD)	22.7 ± 3.2	23.1 ± 4.2	0.528	22.1 ± 3.9	22.8 ± 5.5	0.621
*Helicobacter pylori* infection, *n* (%)						
Infected	22 (31.9)	22 (32.8)	0.716	13 (38.2)	11 (33.3)	0.897
Uninfected	15 (21.7)	18 (26.9)		7 (20.6)	8 (24.2)	
Not examined	32 (46.4)	27 (40.3)		14 (41.2)	14 (42.4)	
Smoker, *n* (%)						
Current smoker	16 (23.2)	15 (22.4)	0.956	6 (17.6)	7 (21.2)	0.506
Past smoker	15 (21.7)	16 (23.9)		5 (14.7)	8 (24.2)	
Nonsmoker	38 (55.1)	36 (53.7)		23 (67.6)	18 (54.5)	
Alcoholics, *n* (%)						
Heavy alcoholics	12 (17.4)	10 (14.9)	0.696	6 (17.6)	5 (15.2)	0.783
Social drinker	57 (82.6)	57 (85.1)		28 (82.4)	28 (84.8)	
Laboratory finding						
Fasting blood glucose (mg/dL)	102 ± 6	100 ± 11	0.767	103 ± 8	102 ± 2	0.551
Total cholesterol (mg/dL)	161 ± 22	156 ± 32	0.614	162 ± 11	151 ± 17	0.324
AST (U/L)	27 ± 9	26 ± 2	0.522	26 ± 8	28 ± 8	0.483
ALT (U/L)	19 ± 3	20 ± 4	0.559	17 ± 18	21 ± 3	0.427
ALP (U/L)	74 ± 15	70 ± 12	0.311	73 ± 9	72 ± 2	0.652
Total bilirubin (mg/dL)	0.7 ± 0.6	0.5 ± 0.3	0.682	0.8 ± 0.1	0.6 ± 0.4	0.531
BUN (mg/dL)	14.1 ± 3.1	13.5 ± 3.7	0.747	14.3 ± 2.6	13.1 ± 5.9	0.829
Creatinine (mg/dL)	1.07 ± 0.8	0.97 ± 0.4	0.299	1.09 ± 0.5	0.98 ± 0.9	0.185
Uric acid (mg/dL)	5.2 ± 1.6	5.0 ± 1.5	0.688	4.9 ± 1.9	4.9 ± 2.1	0.759
Blood pressure						
Systolic	130 ± 16	126 ± 15	0.738	126 ± 19	123 ± 21	0.717
Diastolic	73 ± 12	74 ± 7	0.580	75 ± 18	72 ± 15	0.392
TNM stage ^a^		-	-		-	-
I (IA/IB)	46 (37/9)			23 (19/4)		
II (IIA/IIB)	7 (5/2)			3 (2/1)		
III	10			5		
IV	6			3		
Presence of lymph node metastasis, *n* (%)	15 (21.7)	-	-	7 (20.6)	-	-
Histologic diagnosis, *n* (%)		-	-		-	-
Differentiated	37 (53.6)			18 (52.9)		
Undifferentiated	32 (46.4)			16 (47.1)		
Epstein–Barr virus positivity, *n* (%)	8 (11.6)			4 (11.8)		
Serum tumor marker (>cutoff value/total)			-			-
CA 19-9 ^b^	3 (4.3)	1 (1.5)	0.324	1 (2.9)	0	0.321
CEA ^c^	2 (2.9)	1 (1.5)	0.577	0	0	-
CA 72-4 ^d^	6 (8.7)	2 (3.0)	0.157	2 (5.9)	1 (3.0)	0.573

^a^ According to the American Joint Committee on Cancer Staging Manual (7th edition); ^b^ Cutoff value of >37 U/mL; ^c^ Cutoff value of >7 ng/mL; ^d^ Cutoff value of >4 U/mL.

**Table 2:** Identified metabolites, with changes in their levels in gastric cancer (GC) and receiver operating characteristic (ROC) analysis results.

No.	Metabolites	Chemical Shift (ppm)	*p*-Value	Changes (GC over Controls)	ROC Analysis		
					AUC	Sensitivity (%)	Specificity (%)
1	Alanine	1.49(d),	1.18 × 10^−4^	▲	0.748	70	80
2	Citrate	2.54(d), 2.68(d)	1.60 × 10^−4^	▲	0.632	50	70
3	Creatine	3.04(s)	1.90 × 10^−9^	▲	0.863	80	90
4	Creatinine	3.05(s), 4.07(s)	3.27 × 10^−5^	▽	0.723	60	80
5	Glycerol	3.57(m), 3.66(m), 3.78(m),	7.51 × 10^−19^	▽	0.936	90	90
6	Hippurate	3.98(d), 7.55(t), 7.64(t), 7.83(m)	1.75 × 10^−6^	▲	0.74	70	70
7	Phenylalanine	7.32(m), 7.38(m), 7.42(m)	1.13 × 10^−8^	▲	0.802	80	70
8	Taurine	3.27(t), 3.43(t)	1.90 × 10^−6^	▲	0.759	80	70
9	3-hydroxybutyrate	1.21(d)	4.61 × 10^−4^	▽	0.706	60	70

▲ represents up-regulation and ▽ represents down-regulation.

**Table 3:** Comparison of sensitivity and specificity of conventional modalities and metabolomics profiling for early-stage and locally advanced gastric cancer.

Variable	Metabolomics				Serum Markers		
	All Stages	Stage I + II	Stage I (IA + IB)	Stage IA	CEA	CA 19-9	CA 72-4
Sensitivity (%)	93.9	90.9	97.0	97.0	1.9	3.9	7.8
Specificity (%)	94.1	92.3	95.7	94.7	99.0	99.0	97.0

## Supplementary Materials

The following are available online at https://www.mdpi.com/2072-6694/12/10/2904/s1, Figure S1: *Helicobacter pylori* effects on healthy subjects and patients with gastric cancer, Figure S2: Snapshot of metabolite identification using the Chenomx NMR suite, Figure S3: Cancer stage-dependent prediction of healthy subjects and patients with gastric cancer, Figure S4: Pathway-based network analysis using MetScape and Metaboanalyst. The identified metabolites were loaded into MetScape, and a pathway-based network was built using the compound–reaction–enzyme–gene mode, Figure S5: Levels of differentially expressed genes associated with gastric cancer metabolic markers, Figure S6: Validation of changes in differentially expressed gene levels using an independent microarray dataset, Table S1: Comparison of metabolite concentrations, Table S2: List of genes potentially related to metabolites, Table S3: Supplementary dataset.

## References

[1] Bray F., Ferlay J., Soerjomataram I., Siegel R.L., Torre L.A., Jemal A. (2018). Global cancer statistics 2018: GLOBOCAN estimates of incidence and mortality worldwide for 36 cancers in 185 countries. CA Cancer J. Clin..

[2] Duraes C., Almeida G.M., Seruca R., Oliveira C., Carneiro F. (2014). Biomarkers for gastric cancer: Prognostic, predictive or targets of therapy?. Virchows Arch..

[3] Ludwig J.A., Weinstein J.N. (2005). Biomarkers in cancer staging, prognosis and treatment selection. Nat. Rev. Cancer.

[4] Hallissey M.T., Allum W.H., Jewkes A.J., Ellis D.J., Fielding J.W. (1990). Early detection of gastric cancer. BMJ.

[5] Ono H. (2006). Early gastric cancer: Diagnosis, pathology, treatment techniques and treatment outcomes. Eur. J. Gastroenterol. Hepatol..

[6] Montgomery M., Fukuhara S., Karpeh M., Brower S. (2013). Evidence-based review of the management of early gastric cancer. Gastroenterol. Rep. Oxf..

[7] Pyo J.H., Lee H., Min B.H., Lee J.H., Choi M.G., Lee J.H., Choi M.G., Lee J.H., Sohn T.S., Bae J.M. (2016). Long-Term Outcome of Endoscopic Resection vs. Surgery for Early Gastric Cancer: A Non-inferiority-Matched Cohort Study. Am. J. Gastroenterol..

[8] Yao K., Anagnostopoulos G.K., Ragunath K. (2009). Magnifying endoscopy for diagnosing and delineating early gastric cancer. Endoscopy.

[9] Tanaka M., Ono H., Hasuike N., Takizawa K. (2008). Endoscopic submucosal dissection of early gastric cancer. Digestion.

[10] Layke J.C., Lopez P.P. (2004). Gastric cancer: Diagnosis and treatment options. Am. Fam. Physician.

[11] Tajiri H., Doi T., Endo H., Nishina T., Terao T., Hyodo I., Matsuda K., Yagi K. (2002). Routine endoscopy using a magnifying endoscope for gastric cancer diagnosis. Endoscopy.

[12] Okines A., Verheij M., Allum W., Cunningham D., Cervantes A. (2010). Group EGW. Gastric cancer: ESMO Clinical Practice Guidelines for diagnosis, treatment and follow-up. Ann. Oncol..

[13] Kaise M., Ohkura Y., Iizuka T., Kimura R., Nomura K., Kuribayashi Y., Yamada A., Yamashita S., Furuhata T., Kikuchi D. (2015). Endocytoscopy is a promising modality with high diagnostic accuracy for gastric cancer. Endoscopy.

[14] Kim G.H. (2019). Is Endoscopic Grading of Atrophic Gastritis Useful for Predicting the Risk of Gastric Cancer?. Korean J. Helicobacter Gastrointest. Res..

[15] Guadagni F., Roselli M., Amato T., Cosimelli M., Perri P., Casale V., Carlini M., Santoro E., Cavaliere R., Greiner John W. (1992). CA 72-4 measurement of tumor-associated glycoprotein 72 (TAG-72) as a serum marker in the management of gastric carcinoma. Cancer Res..

[16] Ishigami S., Natsugoe S., Hokita S., Che X., Tokuda K., Nakajo A., Iwashige H.T., Tokushige M., Watanabe T., Takao S. (2001). Clinical importance of preoperative carcinoembryonic antigen and carbohydrate antigen 19-9 levels in gastric cancer. J. Clin. Gastroenterol..

[17] Lai I.R., Lee W.J., Huang M.T., Lin H.H. (2002). Comparison of serum CA72-4, CEA, TPA, CA19-9 and CA125 levels in gastric cancer patients and correlation with recurrence. Hepato Gastroenterol..

[18] Wobbes T., Thomas C.M., Segers M.F., Nagengast F.M. (1992). Evaluation of seven tumor markers (CA 50, CA 19-9, CA 19-9 TruQuant, CA 72-4, CA 195, carcinoembryonic antigen, and tissue polypeptide antigen) in the pretreatment sera of patients with gastric carcinoma. Cancer.

[19] Carpelan-Holmstrom M., Louhimo J., Stenman U.H., Alfthan H., Haglund C. (2002). CEA, CA 19-9 and CA 72-4 improve the diagnostic accuracy in gastrointestinal cancers. Anticancer Res..

[20] Kodera Y., Isobe K., Yamauchi M., Satta T., Hasegawa T., Oikawa S., Kondoh K., Akiyama S., Itoh K., Nakashima I. (1993). Expression of carcinoembryonic antigen (CEA) and nonspecific crossreacting antigen (NCA) in gastrointestinal cancer; the correlation with degree of differentiation. Br. J. Cancer.

[21] Ucar E., Semerci E., Ustun H., Yetim T., Huzmeli C., Gullu M. (2008). Prognostic value of preoperative CEA, CA 199–, CA 724–, and AFP levels in gastric cancer. Adv. Ther..

[22] Yang A.P., Liu J., Lei H.Y., Zhang Q.W., Zhao L., Yang G.H. (2014). CA72-4 combined with CEA, CA125 and CAl9-9 improves the sensitivity for the early diagnosis of gastric cancer. Clin. Chim. Acta.

[23] Jiang Z., Zhang C., Gan L., Jia Y., Xiong Y., Chen Y., Wang Z., Wang L., Luo H., Li J. (2018). iTRAQ-Based Quantitative Proteomics Approach Identifies Novel Diagnostic Biomarkers That Were Essential for Glutamine Metabolism and Redox Homeostasis for Gastric Cancer. Proteom. Clin. Appl..

[24] Simonian M., Mosallayi M., Mirzaei H. (2018). Circulating miR-21 as novel biomarker in gastric cancer: Diagnostic and prognostic biomarker. J. Cancer Res. Ther..

[25] Jin X., Yun S.J., Jeong P., Kim I.Y., Kim W.J., Park S. (2014). Diagnosis of bladder cancer and prediction of survival by urinary metabolomics. Oncotarget.

[26] Wen H., Yoo S.S., Kang J., Kim H.G., Park J.S., Jeong S., Lee J.I., Kwon H.N., Kang S., Lee D. (2010). A new NMR-based metabolomics approach for the diagnosis of biliary tract cancer. J. Hepatol..

[27] Carrola J., Rocha C.M., Barros A.S., Gil A.M., Goodfellow B.J., Carreira I.M., Bernardo J., Gomes A., Sousa V., Carvalho L. (2011). Metabolic signatures of lung cancer in biofluids: NMR-based metabonomics of urine. J. Proteome Res..

[28] Kim K., Aronov P., Zakharkin S.O., Anderson D., Perroud B., Thompson I.M., Weiss R.H. (2009). Urine metabolomics analysis for kidney cancer detection and biomarker discovery. Mol. Cell Proteom..

[29] Qiu Y., Cai G., Su M., Chen T., Liu Y., Xu Y., Ni Y., Zhao A., Cai S., Xu L.X. (2010). Urinary metabonomic study on colorectal cancer. J. Proteome Res..

[30] Chen J.L., Fan J., Lu X.J. (2014). CE-MS based on moving reaction boundary method for urinary metabolomic analysis of gastric cancer patients. Electrophoresis.

[31] Jung J., Jung Y., Bang E.J., Cho S.I., Jang Y.J., Kwak J.M., Park S., Hwang G.S. (2014). Noninvasive Diagnosis and Evaluation of Curative Surgery for Gastric Cancer by Using NMR-based Metabolomic Profiling. Ann. Surg. Oncol..

[32] Liang Q., Wang C., Li B. (2015). Metabolomic Analysis Using Liquid Chromatography/Mass Spectrometry for Gastric Cancer. Appl. Biochem. Biotechnol..

[33] Chan A.W., Mercier P., Schiller D., Bailey R., Robbins S., Eurich D.T., Dean T., Sawyer M.B., Broadhurst D. (2015). 1H-NMR urinary metabolomic profiling for diagnosis of gastric cancer. Br. J. Cancer.

[34] Zhang Y., Ren H., Jiang Y., Gao Y., Liu S. (2012). Urinary metabonomics of stomach cancer assessed by rapid resolution liquid chromatography/time-of-fight mass spectrometry. Chin. Med. J..

[35] Kim E.R., Kwon H.N., Nam H., Kim J.J., Park S., Kim Y.H. (2019). Urine-NMR metabolomics for screening of advanced colorectal adenoma and early stage colorectal cancer. Sci. Rep..

[36] Bylesjo M., Rantalainen M., Cloarec O., Nicholson J.K., Holmes E., Trygg J. (2006). OPLS discriminant analysis: Combining the strengths of PLS-DA and SIMCA classification. J. Chemometr..

[37] Hirayama A., Kami K., Sugimoto M., Sugawara M., Toki N., Onozuka H., Kinoshita T., Saito N., Ochiai A., Tomita M. (2009). Quantitative metabolome profiling of colon and stomach cancer microenvironment by capillary electrophoresis time-of-flight mass spectrometry. Cancer Res..

[38] Chen X., Leung S.Y., Yuen S.T., Chu K.M., Ji J., Li R., Chan A.S.Y., Law S., Troyanskaya O.G., Wong J. (2003). Variation in gene expression patterns in human gastric cancers. Mol. Biol. Cell.

[39] Karnovsky A., Weymouth T., Hull T., Tarcea V.G., Scardoni G., Laudanna C., Sartor M.A., Stringer K.A., Jagadish H.V., Burant C. (2012). Metscape 2 bioinformatics tool for the analysis and visualization of metabolomics and gene expression data. Bioinformatics.

[40] Napoli C., Sperandio N., Lawlor R.T., Scarpa A., Molinari H., Assfalg M. (2012). Urine metabolic signature of pancreatic ductal adenocarcinoma by (1)h nuclear magnetic resonance: Identification, mapping, and evolution. J. Proteome Res..

[41] Slupsky C.M., Steed H., Wells T.H., Dabbs K., Schepansky A., Capstick V., Faught W., Sawyer M.B. (2010). Urine metabolite analysis offers potential early diagnosis of ovarian and breast cancers. Clin. Cancer Res..

[42] Hasim A., Ma H., Mamtimin B., Abudula A., Niyaz M., Zhang L.W., Anwer J., Sheyhidin I. (2012). Revealing the metabonomic variation of EC using (1)H-NMR spectroscopy and its association with the clinicopathological characteristics. Mol. Biol. Rep..

[43] Song H., Wang L., Liu H.L., Wu X.B., Wang H.S., Liu Z.H., Li Y., Diao D., Chen H., Peng J. (2011). Tissue metabolomic fingerprinting reveals metabolic disorders associated with human gastric cancer morbidity. Oncol. Rep..

[44] Wu H., Xue R., Tang Z., Deng C., Liu T., Zeng H., Sun Y., Shen X. (2010). Metabolomic investigation of gastric cancer tissue using gas chromatography/mass spectrometry. Anal. Bioanal. Chem..

[45] Yang T., Luo P., Li Y., Hua R., Yin P., Xu G. (2014). [A serum metabolomics study of gastric cancer based on pseudotargeted liquid chromatography-mass spectrometry approach]. Se Pu Chin. J. Chromatogr..

[46] Wyss M., Kaddurah-Daouk R. (2000). Creatine and creatinine metabolism. Physiol. Rev..

[47] Swaminathan R., Major P., Snieder H., Spector T. (2000). Serum creatinine and fat-free mass (lean body mass). Clin. Chem..

[48] Brindle J.T., Antti H., Holmes E., Tranter G., Nicholson J.K., Bethell H.W.L., Clarke S., Schofield P.M., McKilligin E., Mosedale D.E. (2002). Rapid and noninvasive diagnosis of the presence and severity of coronary heart disease using 1H-NMR-based metabonomics. Nat. Med..

[49] Davis V.W., Schiller D.E., Eurich D., Sawyer M.B. (2012). Urinary metabolomic signature of esophageal cancer and Barrett’s esophagus. World J. Surg. Oncol..

[50] Kandoth C., McLellan M.D., Vandin F., Ye K., Niu B., Lu C., Xie M., Zhang Q., McMichael J.F., Wyczalkowski M.A. (2013). Mutational landscape and significance across 12 major cancer types. Nature.

[51] Abbassi-Ghadi N., Kumar S., Huang J., Goldin R., Takats Z., Hanna G.B. (2013). Metabolomic profiling of oesophago-gastric cancer: A systematic review. Eur. J. Cancer.

[52] Kim K.B., Yang J.Y., Kwack S.J., Park K.L., Kim H.S., Ryu D.H., Kim Y., Hwang G., Lee B.M. (2010). Toxicometabolomics of urinary biomarkers for human gastric cancer in a mouse model. J. Toxicol. Environ. Health Part A.

[53] Kim N., Jung H.C. (2010). The role of serum pepsinogen in the detection of gastric cancer. Gut Liver.

[54] Emwas A.-H., Saccenti E., Gao X., McKay R.T., dos Santos V.A.M., Roy R., Wishart D.S. (2018). Recommended strategies for spectral processing and post-processing of 1D 1 H-NMR data of biofluids with a particular focus on urine. Metabolomics.

[55] Kang J., Choi M.-Y., Kang S., Kwon H.N., Wen H., Lee C.H., Park M., Wiklund S., Kim H.J., Kwon S.W. (2008). Application of a 1H nuclear magnetic resonance (NMR) metabolomics approach combined with orthogonal projections to latent structure-discriminant analysis as an efficient tool for discriminating between Korean and Chinese herbal medicines. J. Agric. Food Chem..

