# Supplementary material for: Screening for Early Gastric Cancer Using a Noninvasive Urine Metabolomics Approach
Source: Cancers (Basel). 2020 Oct 9;12(10):2904. doi: 10.3390/cancers12102904 (PMC7599479; doi:10.3390/cancers12102904)
Supplement: Supplementary file 1 [file cancers-12-02904-s001.zip › second supplementary/cancers-938549-supplementary-second publish.docx]

Screening for Early Gastric Cancer Using a Noninvasive Urine Metabolomics Approach

Hyuk Nam Kwon, Hyuk Lee, Ji Won Park, Young-Ho Kim, Sunghyouk Park and Jae J. Kim


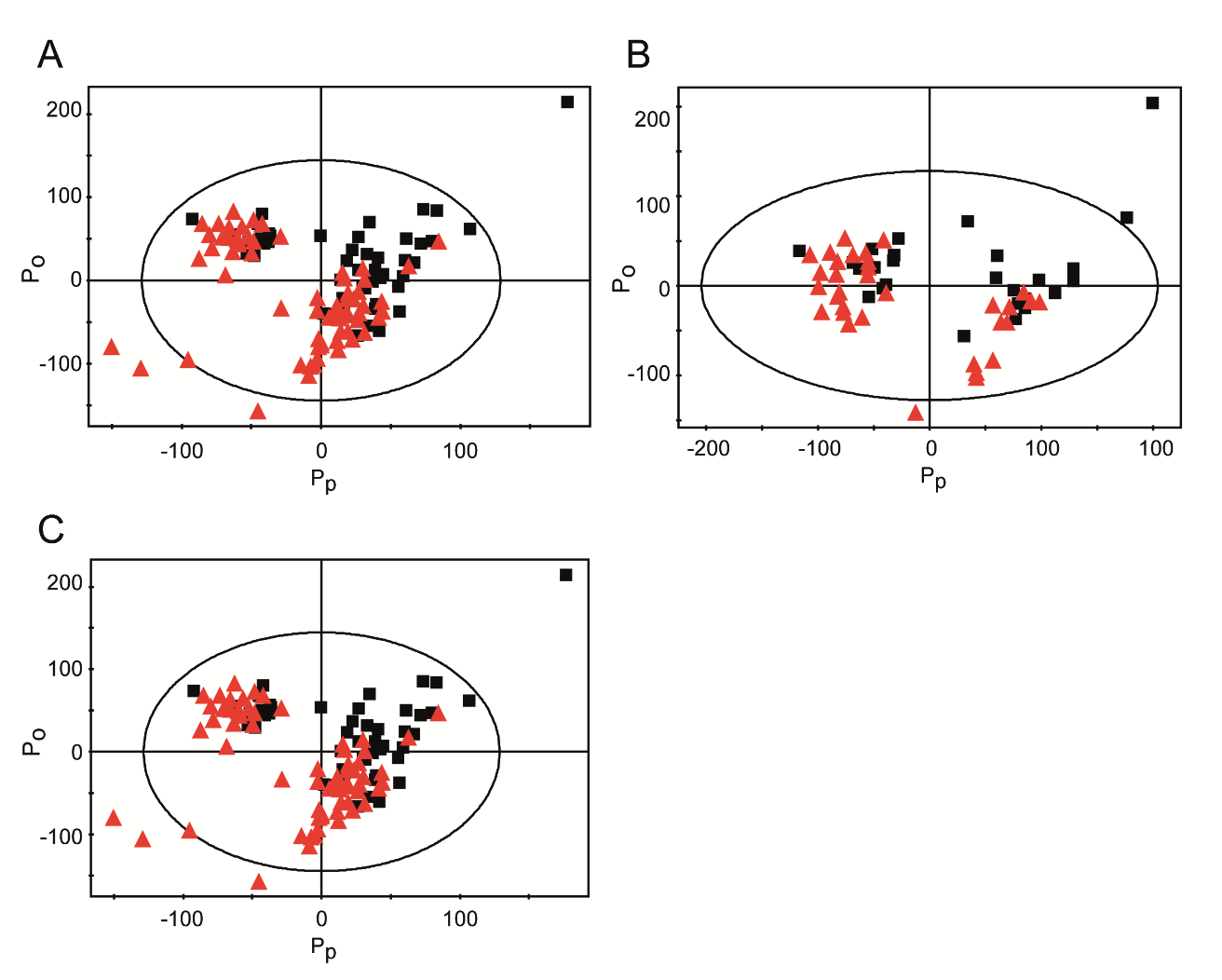


**Figure S1.** *Helicobacter pylori* effects on healthy subjects and patients with gastric cancer. Partial least squares-discriminant analysis (PLS-DA) score plots of (**A**) healthy and all stage gastric cancer subjects, (**B**) healthy subjects, and (**C**) gastric cancer subjects. No significant differences were found based on *Helicobacter pylori* infection.


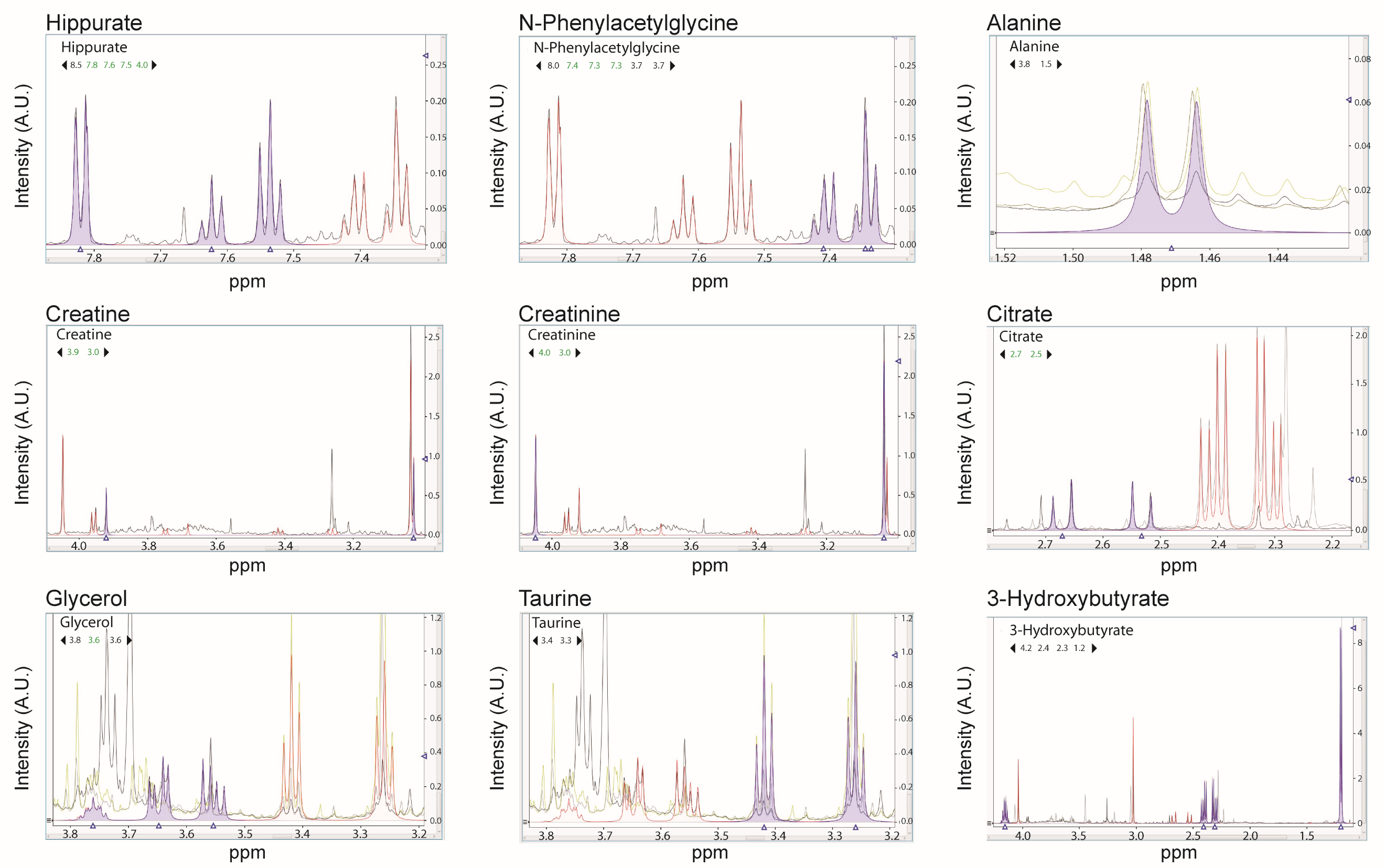


**Figure S2.** Snapshot of metabolite identification using the Chenomx NMR suite.


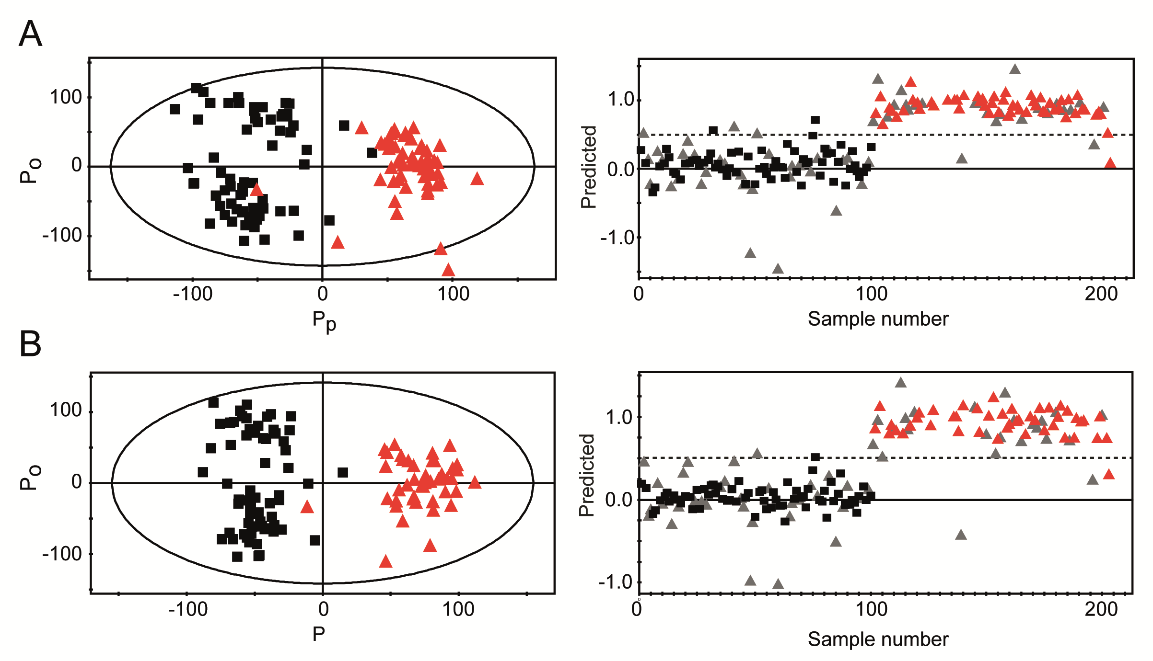


**Figure S3.** Cancer stage-dependent prediction of healthy subjects and patients with gastric cancer. (**A**) The gastric cancer group included stage I (IA + IB) and stage II patients (n = 53), and the OPLS-DA analysis was performed with the healthy control group (n = 67). For validation, 59 unknown samples were fitted into the OPLS-DA model, and the specificity and sensitivity were calculated. (**B**) Patients with stage I (IA + IB) gastric cancer (n = 46) were clustered as the gastric cancer group, and the same statistical analysis was applied versus the same healthy control group (n = 67). Validation was achieved with 56 unknown samples. Black boxes: samples from healthy controls; red triangles: samples from patients with gastric cancer. In the case of misclassified samples, the predicted *Y*-values for unknown samples are shown as gray triangles.


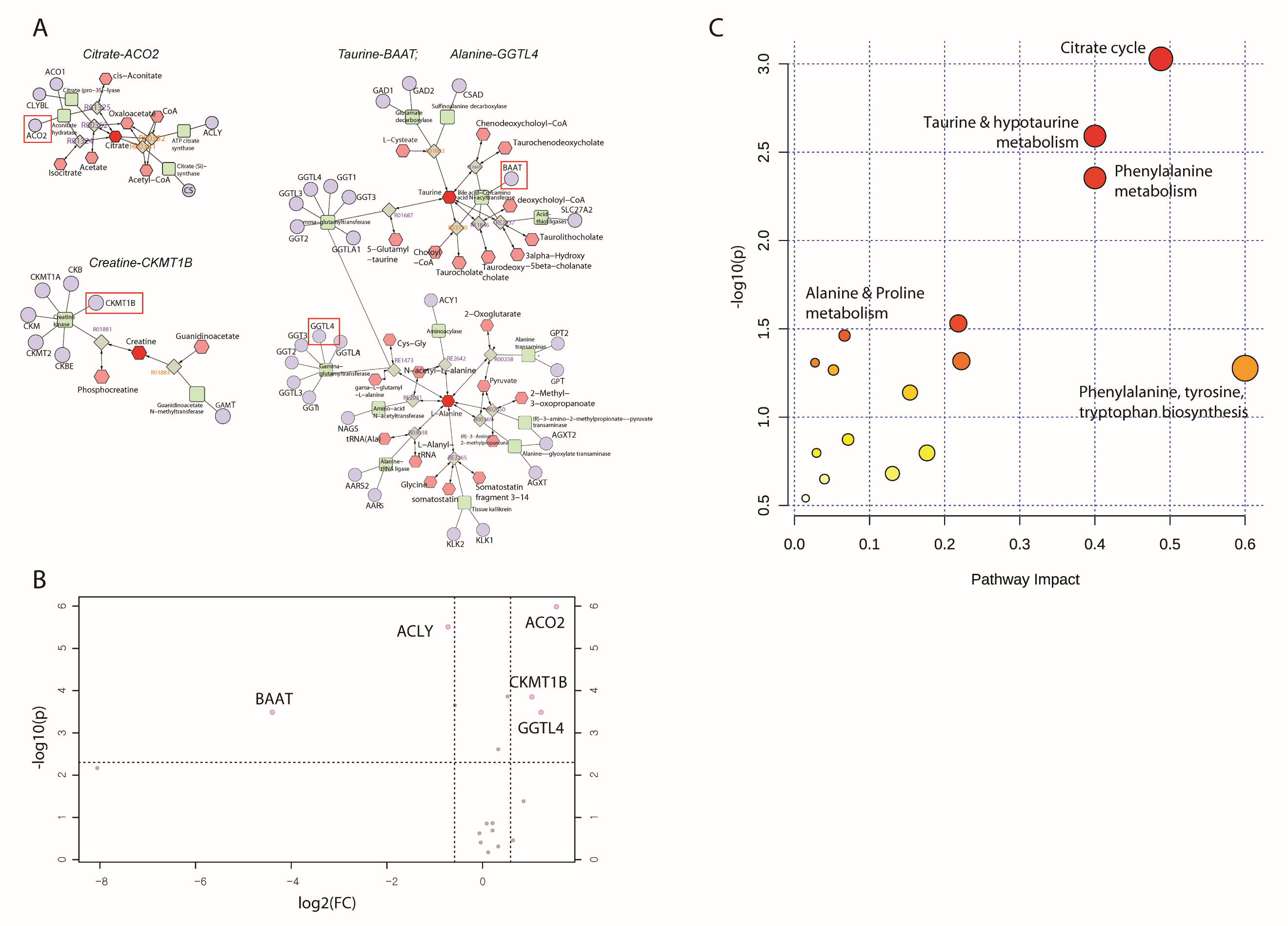


**Figure S4.** Pathway-based network analysis using MetScape and Metaboanalyst. (**A**) The identified metabolites were loaded into MetScape, and a pathway-based network was built using the compound–reaction–enzyme–gene mode. The red hexagon represents the loaded metabolite, and light purple circles refer to the genes potentially associated with the metabolite. Lines with arrows represent the expansion of the network relationships. Red boxes, highlighting selected genes, refer to statistically meaningful changes in a microarray dataset; (**B**) The volcano plot analysis was performed using the 1.5-fold change criterion and a false discovery rate (FDR) of 0.005; five genes were selected; (**C**) Joint pathway analysis through Metaboanalyst. Identified metabolites and correlated genes were used and the major important metabolisms are denoted. *ACLY*: ATP citrate lyase; *ACO2*: aconitase 2; *BAAT*: bile acid coenzyme A; *CKMT1B*: creatine kinase, mitochondrial 1B; *GGTL4*: gamma-glutamyl transferase light chain 2.


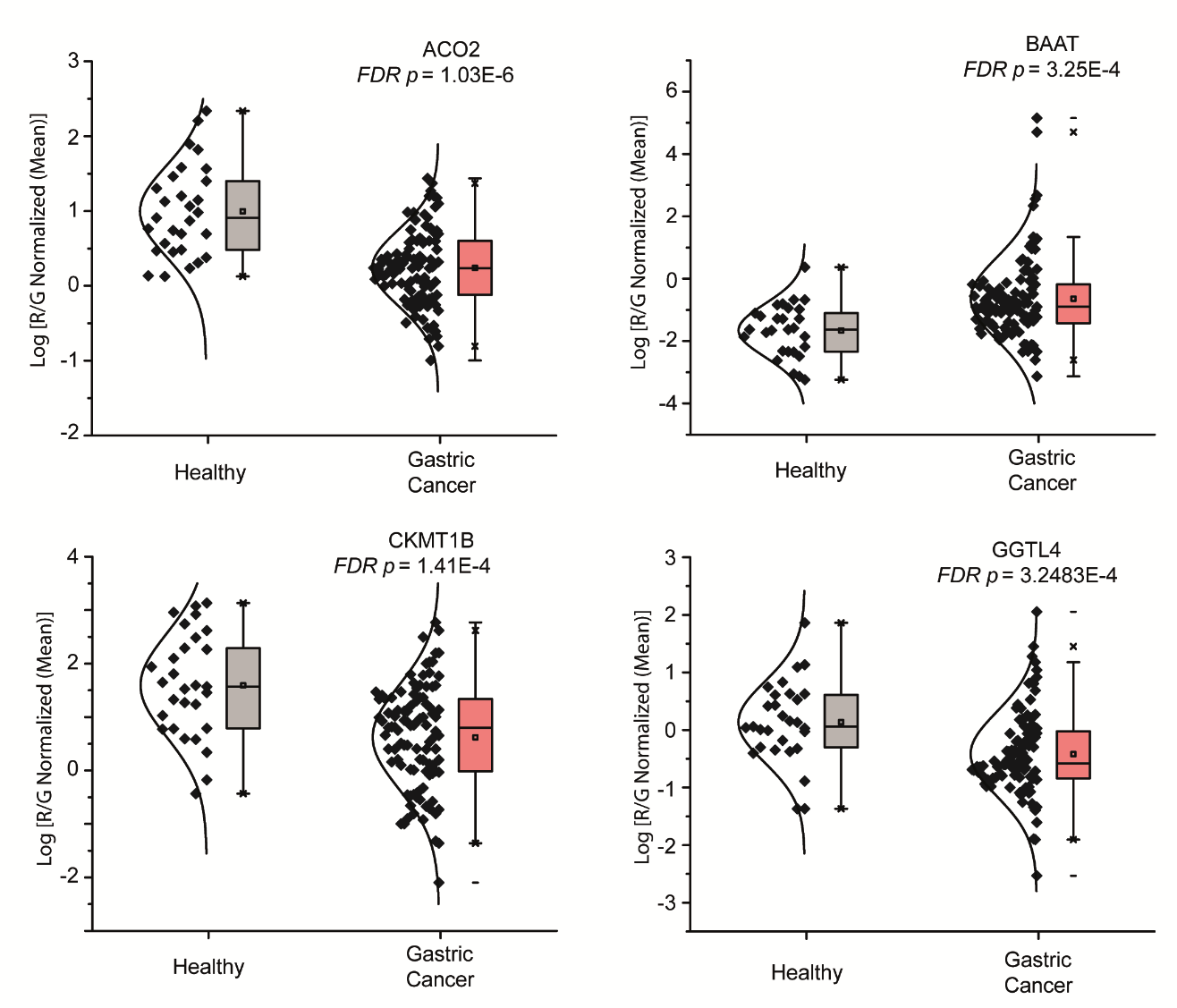


**Figure S5.** Levels of differentially expressed genes associated with gastric cancer metabolic markers. *ACO2*: aconitase 2; *BAAT*: bile acid coenzyme A; *CKMT1B*: creatine kinase, mitochondrial 1B; *GGTL4*: gamma-glutamyl transferase light chain 2.


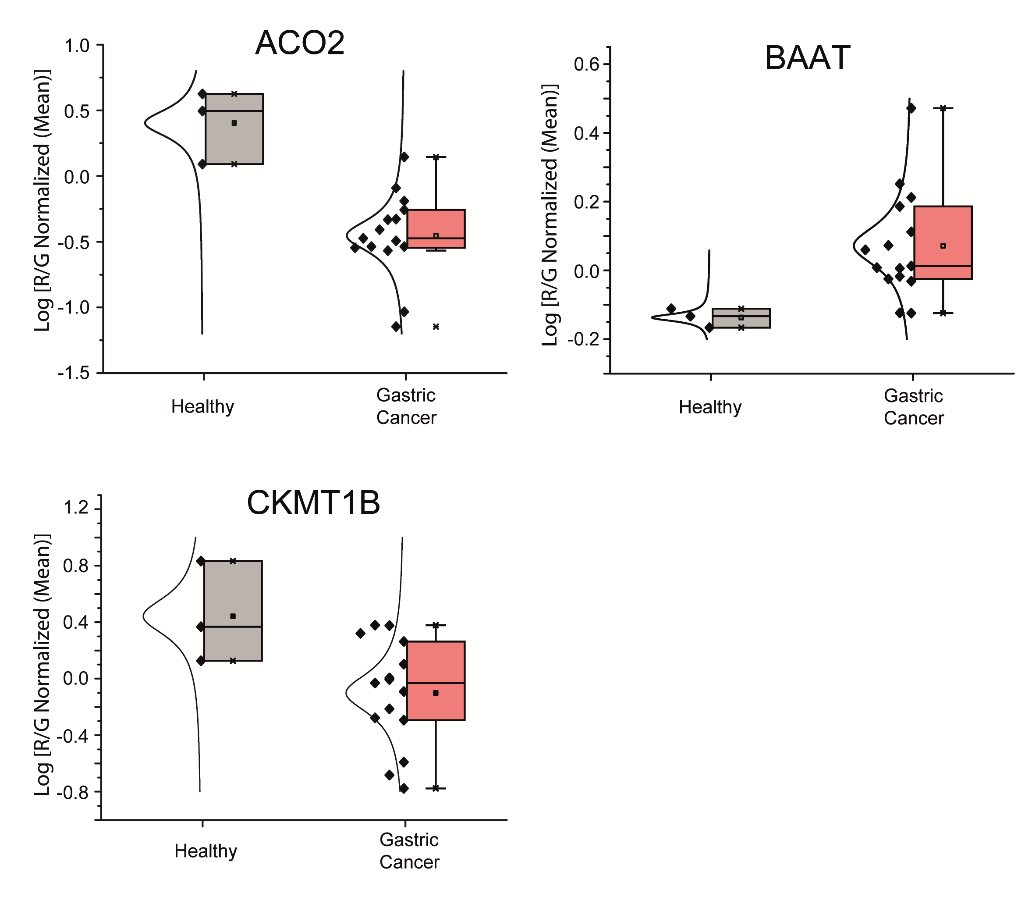


**Figure S6.** Validation of changes in differentially expressed gene levels using an independent microarray dataset. *ACO2*: aconitase 2; *BAAT*: bile acid coenzyme A; *CKMT1B*: creatine kinase, mitochondrial 1B;.

**Table S1.** Comparison of metabolite concentrations.

| Metabolites | Average | | Standard Deviation | | Fold Change  (GC over Healthy) |
| --- | --- | --- | --- | --- | --- |
|  | Healthy | Gastric Cancer | Healthy | Gastric Cancer |  |
| Alanine | 110.5621 | 138.6403 | 34.74577 | 47.08516 | 1.254 |
| Citrate | 311.5533 | 396.2289 | 192.549 | 212.8475 | 1.272 |
| Creatine | 236.0962 | 356.0028 | 57.65586 | 137.5493 | 1.508 |
| Creatinine | 2057.457 | 1232.484 | 1150.17 | 1096.039 | 0.599 |
| Glycerol | 666.7117 | 262.9633 | 273.437 | 101.5127 | 0.394 |
| Hippurate | 17.38305 | 29.33655 | 11.09883 | 16.27456 | 1.687653 |
| Phenylalanine | 86.49263 | 187.7314 | 66.44612 | 118.273 | 2.170491 |
| Taurine | 1120.755 | 2505.729 | 775.844 | 2110.373 | 2.23575 |
| 3-Hydroxybutyrate | 167.7756 | 136.6161 | 55.64541 | 45.49517 | 0.814279 |

**Table S2.** List of genes potentially related to metabolites.

| **Gene** | **FC** | **Log2(FC)** | **Adjusted *p*-Value** |
| --- | --- | --- | --- |
| **AARS2** | 0.96354 | -0.05359 | 0.6236 |
| **ACLY** | 0.60594 | -0.72275 | 2.43E-05 |
| **ACO1** | 1.4365 | 0.5226 | 3.21E-06 |
| **ACO2** | 2.9132 | 1.5426 | 6.81E-09 |
| **AGXT** | 1.7338 | 0.79394 | 0.006852 |
| **AGXT2** | 1.1553 | 0.20825 | 0.13887 |
| **BAAT** | 0.048656 | -4.3612 | 0.000762 |
| **CKMT1B** | 2.0419 | 1.0299 | 2.43E-05 |
| **CKMT2** | 1.2543 | 0.32687 | 0.000762 |
| **CS** | 0.6587 | -0.60231 | 0.27794 |
| **CSAD** | 1.0614 | 0.085914 | 0.40527 |
| **GAD1** | 0.006218 | -7.3293 | 0.000851 |
| **GAD2** | 1.0739 | 0.10281 | 0.6236 |
| **GAMT** | 0.98911 | -0.0158 | 0.84868 |
| **GGTL4** | 2.3271 | 1.2186 | 0.000902 |
| **GPT2** | 1.6108 | 0.68777 | 0.00656 |
| **KLK2** | 1.2087 | 0.27348 | 0.096992 |
| **NAGS** | 1.1437 | 0.1937 | 0.013065 |

*AARS: Alanine-tRNA ligase; ACLY*: ATP citrate lyase; *ACO1*: aconitase 1; *ACO2*: aconitase 2; AGXT: Alanine-glyoxylate aminotransferase; AGXT2: Alanine-glyoxylate aminotransferase 2; *BAAT*: bile acid coenzyme A; *CKMT1B*: creatine kinase, mitochondrial 1B; CKMT2: Creatine kinase S-type; CS: Citrate synthase; *GAD1*: glutamate decarboxylase 1; *GAD2*: glutamate decarboxylase 2; GAMT: Guanidinoacetate N-methyltransferase; *GGTL4*: gamma-glutamyl transferase light chain 2; GPT2: Glutamic pyruvic transaminase 2; KLK2: Kallikrein 2 precursor; NAGS: N-acetylglutamate synthase.

| 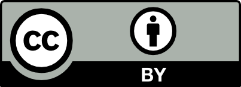 | © 2020 by the authors. Licensee MDPI, Basel, Switzerland. This article is an open access article distributed under the terms and conditions of the Creative Commons Attribution (CC BY) license (http://creativecommons.org/licenses/by/4.0/). |
| --- | --- |
